# Activity Recognition Using Wearable Physiological Measurements: Selection of Features from a Comprehensive Literature Study

**DOI:** 10.3390/s19245524

**Published:** 2019-12-13

**Authors:** Inma Mohino-Herranz, Roberto Gil-Pita, Manuel Rosa-Zurera, Fernando Seoane

**Affiliations:** 1 Department of Signal Theory and Communications, University of Alcala, 28805 Alcala de Henares, Madrid, Spain; roberto.gil@uah.es (R.G.-P.); manuel.rosa@uah.es (M.R.-Z.); 2 Clinical Science, Intervention an Technology, Karolinska Institutet, 17177 Stockholm, Sweden; fernando.seoane@ki.se; 3 Department Biomedical Engineering, Karolinska University Hospital, 14186 Stockholm, Sweden; 4 Swedish School of Textiles, University of Boras, 50190 Boras, Sweden

**Keywords:** activity recognition, physiological signals, electrocardiogram, thoracic electrical bioimpedance, electrodermal activity

## Abstract

Activity and emotion recognition based on physiological signal processing in health care applications is a relevant research field, with promising future and relevant applications, such as health at work or preventive care. This paper carries out a deep analysis of features proposed to extract information from the electrocardiogram, thoracic electrical bioimpedance, and electrodermal activity signals. The activities analyzed are: neutral, emotional, mental and physical. A total number of 533 features are tested for activity recognition, performing a comprehensive study taking into consideration the prediction accuracy, feature calculation, window length, and type of classifier. Feature selection to know the most relevant features from the complete set is implemented using a genetic algorithm, with a different number of features. This study has allowed us to determine the best number of features to obtain a good error probability avoiding over-fitting, and the best subset of features among those proposed in the literature. The lowest error probability that is obtained is 22.2%, with 40 features, a least squares error classifier, and 40 s window length.

## 1. Introduction

Activity can be defined as the state or quality of being active, which implies that the activity can be emotional, intellectual, physical, etc. Typical activity recognition systems focus on daily life activities such as walking, running, exercising, scrubbing and cooking [1,2,3,4,5], mental tasks [6,7] or emotion recognition [8]. Activity-state recognition systems can be applied to human error prevention tasks in many professional activities such as first responders, crane operators or train drivers. The present work aims at deeply studying several features found in the literature to characterize the signals of electrocardiogram, thoracic bioimpedance and electrodermal activity, whose objective is to recognize four different activities: emotional, mental, physical and neutral activity (resting).

Currently, there are different methods for detecting activity. For instance, Inertial Measurement Units (IMUs) [9,10] in combination with Global Positioning System (GPS) data for outdoor applications [11] or sensor located indoors for smart homes [3,12] for detecting physical activity. On the other hand, speech and gestures can be useful for assessing emotional activity [13,14,15,16]. Another alternative is physiological signals captured through sensors located in the body of the subject. Wearable biomedical sensing through smart clothing [17,18] allows the recording of physiological measurements such as the Electrocardiogram (ECG), the Thoracic Electrical Bioimpedance (TEB) or the Electrodermal Activity (EDA), among others, which contain not only information about specific body functions and physiological states, but also valuable information about the activity and the person’s condition regarding emotional state, mental load and physical activity [19].

In the literature, numerous works are found in which these three signals are used to detect stress, emotions, and activity. For instance, ECG is affected by these factors, since the heart rate is directly related to the body and mind condition [20,21,22]. In this sense, the Heart Rate Variability (HRV) has been widely used to extract information about the status of the autonomous nervous system and emotions [23]. On the other hand, TEB can be used as an indicator of the breathing function, and it has been used in different studies for activity recognition [24] and stress detection [25]. EDA measures the activity of sweating glands on the skin which are directly controlled by the sympathetic nervous system, and thus can also be used for emotion recognition [26,27,28,29].

However, few papers provide deep studies including all these three signals with the same purpose, comparing the physiological signals under study and determining which physiological signal provides more relevant information about the individual activity. For instance, the features extracted from TEB signal acquired together with the ECG and the heart sound can be used to study cardiovascular reactivity during emotional activation in men and women [24]. Numerous features have been found for this purpose in the literature, but there is not a clear rule of which ones are more relevant for a given problem. In general, the larger the number of features, the greater the generalization problems, that is, the ability to handle unseen data [30]. Selecting a subset of features results mandatory for many activity recognition application.

Taking all this into account, the present paper aims at assessing the utility of features extracted from ECG, TEB, and EDA in activity recognition systems. These physiological signals have been recorded using sensorized garments combined with wearable instrumentation. We intend to recognize four different activities: emotional activity, mental activity, physical activity, and resting. The paper is structured as follows: Section 1 introduces the problem tackled in this paper; Section 2 is a review of the literature about physiological sensing, window length, features, and possible classifiers; Section 3 summarizes the sensors used to acquire the signals and the mental activity states that are considered; Section 4 presents the experiments carried out; Section 5 includes the obtained results; Section 6 presents the main conclusions. A set of Appendix A, Appendix B and Appendix C are also included with a detailed description of the considered features extracted from the different acquired signals.

## 2. Background

In this study several parameters have been analyzed: (a) the physiological sensing mode (ECG, TEB and EDA), (b) the window length, (c) the features extracted from each signal, (d) the number of features to obtain the best results, and (e) the type of classifier.

- Selection of Physiological sensing modality: In this part, we compare the physiological signal under study and determine which physiological signal provides more relevant information about the individual activity. The signals used are ECG, TEB, and EDA. It is possible to find numerous works in which these signals are used to detect stress, emotions, and activity in the literature. The ECG signal is used in some papers such as [23], where the obtained results suggest that positive emotions lead to alterations in HRV, which may be beneficial in some illness treatment [19,31,32].
  TEB is also used in some papers, though it is less useful than ECG and EDA signals. The work [25] demonstrated that its use is decisive to detect stress. In addition, most of the studies considered several signals, such as the paper [28] which contains the study on the correlation between heart rate, electrodermal activity and Player Experience in First-Person Shooter Games, concluding that their results indicate correlation between the physiological measures and gameplay experience, even in relatively simple measurement scenarios. Another work, [29] studies the individual differences within the electrodermal activity as subjects’ anxiety, which concludes that in normal subjects there are individual electrodermal differences as a function of trait-anxiety scores. However, few papers provide a deep study of features for the three signals, such as the use of these signals with the same purpose.
- In order to obtain the window length, the first limit found in the literature review is imposed by feature calculation. There are some features that require a minimum window length to be calculated, such as, HRV triangular index, which takes at least 20 min to be calculated [33,34,35], Standard Deviation of NN intervals (SDNN) index, calculated as mean standard deviations of all NN intervals for all 5 min segments of the entire recording [34], and for all derivatives (Standard Deviation of Successive Differences (SDSD), Standard Deviation of sequential 5-min RR interval (SDANN)) found in [34]. In our case, we decided to use window lengths lower than 60 s, as the database could be largely cut down, which would change the study.
- In our study, we have studied a large number of features which have been selected from a deep revision of the literature. The most frequently used with ECG signals were obtained both in the frequency domain and the time domain: frequency bands [23,26,34,36,37,38,39,40,41,42,43,44,45,46,47,48], and power ratios [23,43,44,46,47,49], in frequency domain; and Heart Rate Variability (HRV) [23,26,38,39,41,42,45,48,50,51], the SDNN [42,48,49], Number of NNs in 50 ms (NN50), pNN50 [34,42,48] and some statistical parameters, such as mean amplitude rate, mean frequency, standard deviations of the raw signals, [25,37,52,53,54,55]. In our study, we have studied all the features found in a literature review of more than 90 papers.
  The features extracted from the TEB signal are used in some works such as, [24] where the approach is to study cardiovascular reactivity during emotional activation in men and women. Here, the TEB has been acquired together with ECG and the heart sound. In [56] the full respiratory signal was derived from the thoracic impedance raw data, like in our case.
  Finally, the EDA signal is studied in several papers, [26,42,49,53,55,57,58]. A complete study about the EDA signal is shown in [41,59].
- Most published papers use the calculated features to feed the classifier. Therefore, the number of features used depends on the particular study. We propose to implement feature selection from all the available features to find the best ones and to avoid generalization problems in classification.
- The classifier is usually determined by the author without comparisons or detailed studies about suitability. In numerous works, the selected classifier is the Support Vector Machine (SVM). We think it is positive to make a comparison of different classifiers with very different characteristics.

## 3. Materials

**Figure 1:**
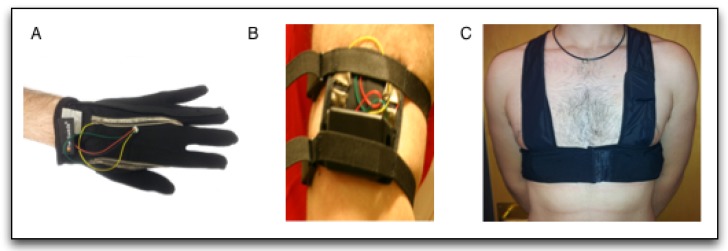
Recording Devices: (**A**) Electrocardiogram (ECG) and Thoracic Electrical Bioimpedance (TEB) device, (**B**) electrodermal activity (EDA) device. (**A**) Glove to acquire EDA signal in hand; (**B**) Arm bracelet to acquire EDA signal. (**C**) Vest to acquire ECG and TEB signals prior published in [25] under license CC by 4.0.

**Figure 2:**
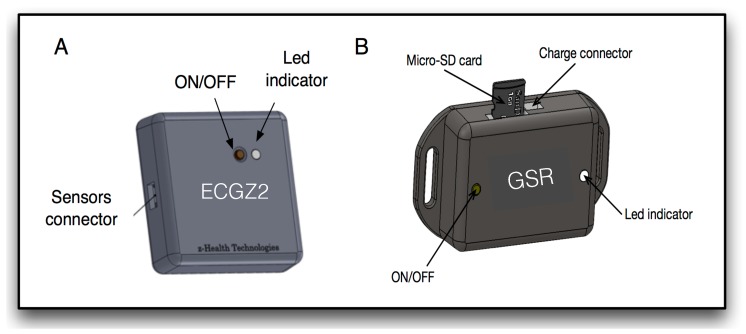
Devices: (**A**) ECGZ2 device, (ECG and TEB recorder prior published in [25] under license CC by 4.0); (**B**) GSR device (EDA recorder published in [60] under license CC by 4.0).

A sensor network capable of acquiring the ECG, TEB and EDA signals has been designed, in order to obtain a database of signals to be used in this study. The complete system acquires all the signals described in the literature, that have been mentioned above, which is explained in detail in [25,60]. To acquire the multimodal biosignals a set of sensorized garments were used, which are shown in Figure 1. A glove to acquire EDA measurement in hand, a bracelet to acquire EDA measurement in the arm and a vest to acquire ECG and TEB. These garments are connected to the measurement devices shown in Figure 2. The glove and the bracelet are connected to the device called GSR, which acquires the EDA signal and the vest is connected to the vest through a recorder called ECGZ2, which acquires ECG and TEB signals. The ECGZ2 is capable of sampling each signal with a different sampling frequency. For the TEB and EDA, the sampling frequency is 100 Hz and for the ECG, the sampling frequency is 250 Hz.

Measurements were collected from k=40 subjects, students and climbers aged 20 to 49, including 12 females and 28 males. The total duration of the complete experiment was approximately 90 min per subject. All of the experiments were performed under the conditions of respect for individual rights and ethical principles that govern biomedical research involving human beings, and written informed consent was obtained from all participants. The experiments were approved by the Research Ethics Committee at the University of Alcala, and the protocol number assigned to this experiment is CEI: 2013/025/201130624.

As was stated above, there were four different activities to be recognized: emotional activity, mental activity, physical activity, and neutral activity (resting).

In order to elicit the different activities, we have used a segment documentary called “Earth” to induce Neutral Activity. In order to elicit emotional activity, we used a set of segments extracted from several validated movies [61]. “American History X” (1998) by Savoy Pictures [62], “I am legend” (2007) by Warner Bross [63], ”Life is beautiful” (1997) by Miramax [64,65] and “Cannibal Holocaust” (1980) by F.D. Cinematografica [66]. The mental activity was elicited using a set of games based on mental arithmetic and playing the well-known game “Tetris”, used several times to elicit mental activity [67].

The designed activity recognition system had to take a decision every 10 s, and each individual generated 28 time slots of each activity (the database is balanced). Thus, the total number of patterns (decisions) for this analysis was 4480, and each class is composed of 1120 different patterns.

In the present analysis, we have used four different activities:

- Neutral activity, registered during the last 140 s of the first movie (the documentary). As each individual watched each movie twice, there are 280 s for each individual in the database.
- Emotional activity, registered during the viewing of the last 70 s of the second and third movies (140 s); therefore, we obtained a total of 280 s per individual.
- Mental activity, registered during the last 140 s of both games, producing 280 s in total.
- Physical activity registered during the last 280 s of the physical activity stage. To elicit physical load the participant had to go up and down the stairs for five minutes.

The database particular characteristics can be found in [25]. The full dataset can also be downloaded from the Supplementary Information included in the paper.

## 4. Methods

The main objective is to extract or calculate all the features found in the literature, applied in different experiments related to activity detection, and after that, to apply a feature selection algorithm to determine the most suitable feature set and the number of features. The acquired signals are processed to identify the activity. The process can be divided into three stages: (a) Feature Extraction, (b) Feature Selection and (c) Classification. An extensive literature review was carried out to find out the typical features used to determine the subject’s activity condition identifying a total of 533 features.

**Figure 3:**
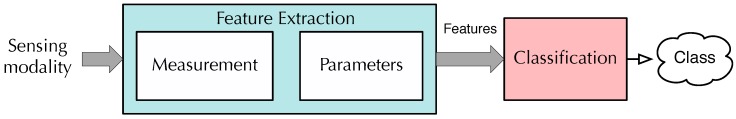
Scheme of the used detection system.

Figure 3 shows the main scheme of the activity recognition system.

### 4.1. Feature Extraction

This stage is divided into two sub-stages. The first one carries out time or frequency domains measurements. These measurements can be the signal acquired itself, or preliminary data used to calculate the features. The second one extracts parameters from each measurement with information related to the classification problem.

The measurements are very dependent on the type of signal. For clarity sake, the description of the measurements and parameters strictly related to a given signal is included in the Appendix A, Appendix B and Appendix C. On the other hand, some parameters are common to all the measurements considered in this work, such as the most common statistical parameters. The statistical parameters considered in this work are denoted as the Standard Set of Statistical Parameters (SSSP), and they include: mean, median, standard deviation, 25% trimmed mean, skewness, kurtosis, maximum, minimum, percentile 25%, percentile 75%, geometric mean, harmonic mean and mean absolute deviation.

In addition to these parameters, another parameter has been frequently calculated in almost all the measurements, which tries to model a very important concept in physiological signal analysis: the baseline. To determine the baseline of a measurement under study, we will use an ultra-low pass filter, so that it integrates the average valued of the measurement over a large period of time. The calculation of this baseline is based on the use of an Infinite Impulse Response (IIR) filter, which can achieve a very low cutoff frequency with only a couple of coefficients. Thus, for a given measurement $z_{i}$, the baseline $y_{i}$ is calculated as follows:

(1) $$y_{i}=z_{i}\cdot \beta +y_{i-1}\cdot \left(1-\beta \right).$$

The β value controls the speed of variation of the baseline parameter, that is, the cutoff frequency of the equivalent low pass filter. Depending on the sampling frequency, we have chosen a value of β which corresponds to a filter that takes approximately the last 20 min of recording of the measurement to obtain the baseline.

Due to the huge number of features, and so as to avoid distractions about the paper goals, the description of the calculated features has been included in a set of Appendix A, Appendix B and Appendix C at the end of this paper.

### 4.2. Classification

The literature of activity recognition using physiological signals includes numerous types of classifiers with different characteristics in terms of complexity, intelligence, and generalization. In this work, we compare the performance of four widely used classifiers with different rules aiming at studying the performance of the set of features: the Least Squares Linear Classifier (LSLC); the Least Squares Quadratic Classifier (LSQC); the Support Vector Machines (SVMs), the Multi-layer Perceptrons (MLPs), the *k*-Nearest Neighbor (kNN), the Centroid Displacement-Based k-Nearest Neighbor (CDNN) and Random Forests (RF).

#### 4.2.1. Least Squares Linear Classifier (LSLC)

In a linear classifier, given a set of training patterns $x={\left[x_{1},x_{2},\ldots ,x_{L}\right]}^{T}$, where each pattern has associated a class, denoted as $C_{i}$, i=1,…,M, the decision rule is obtained using a set of *M* linear combinations of the training patterns. In the least squares approach (the LSLC), the values of the weights of the linear combinations are those that minimize the mean squared error (MSE), obtaining the *Wiener-Hopf* equations [68]. These classifiers are fast and simple, and they present a good generalization capability.

#### 4.2.2. Least Squares Quadratic Classifier (LSQC)

Like with the LSLC, the LSQC also renders very good results with a very fast learning process. It slightly increases the intelligence of the LSLC by adding quadratic terms to the linear combinations, thus improving the performance by increasing the complexity, with the consequence of a decrease in generalization.

#### 4.2.3. Support Vector Machines (SVMs)

An SVM projects the observation vector x to a higher dimension space, using a set of kernel functions, where the patterns can be better linearly separated. The patterns of the design set selected to be the center of these functions are denominated “support vectors” [69]. In the present study, we used linear SVM (LINSVM) and nonlinear SVM using Gaussian Radial Basis Function (RBF) kernels, denoted RBFSVM.

SVMs are essentially binary classifiers, and to implement multi-class classifiers an strategy must be defined. In this paper we used a one-against-all strategy. Furthermore, SVMs present mainly two parameters (the kernel scale and the box constraint) that must be optimized. In this paper a *k*-fold cross validation strategy over the design set was carried out in order to determine the best values of these hyper-parameters. RBFSVMs are also sensitive to differences in the scaling of the features, thus to avoid scale problems features were normalized by removing the mean value and dividing by the standard deviation, being these values estimated using the design data.

#### 4.2.4. Multi-Layer Perceptrons (MLPs)

MLPs are composed of one or more layers of neurons/perceptrons arranged sequentially so that the outputs of the neurons of a layer are the inputs of the neurons of the next layer. It is a feed-forward network, therefore the outputs of the network can be calculated as explicit functions of inputs and weights. Each neuron implements a linear combination of its inputs applied to a nonlinear function denominated activation function. The complexity of the MLP depends on the number of neurons in the hidden layers, allowing to easily control the intelligence of the classifier.

In this paper we considered MLPs with one hidden layer of 8, 12 and 16 neurons. They were trained with the Levenberg Marquardt algorithm, and 20% of the design data was used to monitor and early-stop the training process, avoiding overfitting.

#### 4.2.5. *k*-Nearest Neighbor (kNN)

The kNN is a classification method in which no assumptions are made on the underlying data distribution in the learning process [70]. This classifier estimates the value of the posterior probability in x using the *k* closest patterns from the design database, being *k* a hyper-parameter of the classifier. So, a test pattern x is assigned to the class $C_{i}$ that maximizes the posterior probability, that is, its class is determined by majority voting over the classes of its *k* nearest neighbors. To define the proximity a distance must be defined. In this paper we consider the euclidean distance. To determine the best value of *k* in each case, a *k*-fold validation process was carried out over the design data, and the value of *k* that renders the lowest error rate over the *k*-fold process is selected as the final *k* value. Data from the individuals included in the design set were used as folds on the process.

Like in the case of the RBFSVM, the distance measurement is sensitive to changes in the scale of the features. Thus, features were normalized using the mean and the standard deviation of the features over the design set. Some advantages of the kNNs are: there are no assumptions about data, and it is an easy to understand algorithm. The disadvantages of this classifier include: high memory requirements, computationally expensive, and sensitive to irrelevant features.

#### 4.2.6. Centroid Displacement-Based k-Nearest Neighborgs (CDNN)

The CDNN is a modified version of the kNN algorithm proposed in [71] that replaces the majority voting scheme of the kNN by a centroid based classification criterion. Considering the *k*-th nearest patterns in the database, the centroid of the patterns of each class with and without including the test pattern are evaluated, and the class that suffers less change due to the inclusion of the test pattern is selected. Like in the kNN method, the value of *k* is a hyper-parameter that must be properly determined. Again, *k*-fold cross validation over the design data is used to estimate the best value of *k*. Features were also previously normalized.

#### 4.2.7. Random Forests (RFs)

RFs [72] are classifiers consisting of a collection of *T* tree-structured classifiers $h_{T}\left(x\right)$, k=1,…,T where the decision is taken by majority voting over the *T* independent tree classifiers. Randomization is used in the design of each tree by two factors: first, design data is randomly selected without replacement from the data from the design set. Second, in each node of the tree a subset of *F* features is randomly selected. In this work we grew the trees using CART methodology without pruning, and the ratio of considered features in each node was $F=\lfloor log_{2}M+1\rfloor$, as proposed in [72]. A total of T=200 trees were used to generate each RF classifier.

### 4.3. Feature Selection

Feature selection is the process of selecting a subset of the most relevant features. There are mainly two reasons to use feature selection: to reduce the generalization problems by reducing overfitting and to simplify the model. The feature selection process used in the present work follows the wrapper approach [73]. This approach selects the subset of features that minimize the error rate of a predetermined classification algorithm.

In the literature there are numerous algorithms to select the best features of a set, being Genetic algorithms (GAs) widely used. GAs, proposed in [74], combine the principles of survival of the fittest applying evolutionary laws and emulating biological evolution in nature. These algorithms work with a population consisting of several possible solutions to the problem, being each one of them called chromosome. The optimization is carried out applying modifications to the genes of the chromosomes in the population of possible solutions. They constitute a meta-heuristically search algorithm which can be applied to optimization issues in different areas [75], and they can be successfully applied to the problem of feature selection [76,77].

In our problem, we seek the best reduced set of features which is able to obtain the minimum error probability of a classifier. For this purpose, a “population” of possible sets of features is evaluated with the goal of minimizing the classification error probability, with a limited number of features (the number of selected features must be lower than $N_{max}$). To avoid loss of generalization of the results, the design set is exclusively used to determine the best subset of features by applying a GA, that is, the classification rate optimized by the GA is determined exclusively with the design data.

Since the GA requires the evaluation of many classifiers in the optimization process, the choice of the classifier used in the optimization is crucial. We must consider that for each chromosome in each generation the classifier must be fully trained. Thus, the use of classifiers with a very fast learning process is required. In this work, we rely on the LSLC.

The full process is described as follows:

- A “population” of 100 combinations of features (chromosomes) is randomly generated.
- If there are two combinations with exactly the same set of features, one of them is modified by randomly replacing one of the features.
- For each combination in the population, if the number of features is greater than the maximum $N_{max}$, then features are randomly removed from the chromosome until the condition is satisfied.
- Each combination is ranked using the mean squared error of a LSLC measured using the design set.
- The best 10 combinations of the population are selected as “parents” that survive and are used to regenerate the remaining 90 chromosomes using a random crossover of the parents.
- Mutations are added to the population by changing a feature with a probability of 1%. It is important to highlight that the best individual of each population remains unaltered. The process iterates in Step 2 until a given number of generations are evaluated.

To achieve less risk of premature stalling of the search, we used a method known as Elimination Tournament of GAs [78], that combines several small GAs in a tournament in which the original population of each GA is generated by a random crossover of the “winner” chromosomes from previous GAs.

For this work, the number of features selected was discretized by group size in 5, 10, 20, 40, 60, 80 and the full set, for instance 174 features in case of using the ECG measurement.

To avoid overfitting in the results (generalization loss) while maximizing accuracy in the estimation of the classification error rate, *k*-fold cross-validation was used in the experiments, being k the number of subjects available in the design database, 40 subjects. Thus, the data were divided into *k* folds or subsets containing data from each subject, and each time, the registers from one given subject are used as a test set, with the data from the remaining k−1 used for the design task. For each fold, the design process is carried out, including the feature selection process, the choice of the parameters and the training of the classifier. That is, for each fold, features are normalized estimating the mean and standard deviation of the design set (the remaining k−1 folds in the dataset), the GA is implemented selecting the best subset of features, the classifier is trained with the corresponding methodology, and the hyper-parameters of the classifiers are estimated (please note here that the hyper-parameters were estimated using exclusively the design set). Once this process is completed, the estimated mean and standard deviation is used to normalize the features selected by the GA, and the classifier is evaluated with the previously determined hyper-parameters. The classification of error is then estimated by analyzing the ratio of patterns wrongly classified in the test fold.

The final classification error rate is estimated by averaging the error rates obtained for all the *k* folds. Since data from the same subject is not used for designing and testing at the same time, this method guarantees the generalization of the results to subjects different from the ones included in the database.

This whole process is also repeated 20 times to analyze the statistical significancy of the results. So, the error rate measures the average ratio of classification errors over 40 different test folds (40 individuals of the dataset) and 20 full repetitions of the design process (including feature selection and training the classifier). To study the significance of the results we also carry out a hypothesis test, where the null hypothesis is that the method with the lowest error rate (taken as reference) is not really better than the considered method. So, the performance obtained with different methods and parameters is statistically compared using a single-tail paired-sample t-test over the estimated errors. From this t-test we measure the *p*-value, which can be defined as the level of marginal significance within the statistical hypothesis test [79]. This value represents the probability of obtaining an equal result to or “more extreme” result to what was actually observed when the null hypothesis is true. It is a number between 0 and 1, so that the null hypothesis is rejected if the significance level of the test is less than the significance level (α), which is normally 0.05. The method has been interpreted as follows:

- A small value of *p*-value (typically ≤0.05) implies that the test suggests that the observed data is inconsistent with the null hypothesis, so the null hypothesis must be rejected.
- The hypothesis is not rejected when the p-value is greater than 0.05. This does not imply that the null hypothesis should be accepted, but that it is feasible.

## 5. Results and Analysis

This section includes the analysis of the results obtained in the experiments described in the previous section, including a detailed study of the window length selection, the classifier, the combination of signals, the number of features and the most selected features.

### 5.1. Window Length Selection

The first parameter to determine is the window length. In order to analyze the performance of the system with different window sizes, we consider windows of 10 s, 20 s, 40 s, and 60 s. Please note here that the shift between decisions is fixed in 10 s, independently of the window length. It means that the size of the database and the number of decisions are not affected by the variation in the window length. To determine which window length is the most appropriate to extract the features, several experiments were carried out for each feature set. Table 1 shows the results obtained using the simplest classifier (LSLC) for the different signal combinations considered in this work, as function of the window length. The table includes the best error probability and the number of selected features $N_{max}$ that generates this result. To assess the significance of the results obtained with respect to the window length, the *p*-value [79] has also been included in the table, comparing the best result and the remaining of results for each combination of signals.

The results indicate that the window length for which the obtained error probability is the lowest one is 40 s for all the cases in which the TEB signal is used. We observe that for the ECG signal, the best result was obtained with a window length of 60 s, and for EDA of 10 s. In case of using all the signals, the best result is obtained with a window length of 40 s as well. For this reason, we have fixed the window length to 40 s.

### 5.2. Classifier Selection

To select the best classifier, we have trained the different types of classifiers with different combinations of signals, and a different number of maximum features to be selected. Table 2 contains the error probability (%) obtained for each classifier using the different combination of signals. The best combination of signals is the case including all the physiological signals (ECG+TEB+EDA) with $N_{max}=40$ features, obtaining a 22.2% of error rate, and the second best is the case including ECG and TEB with $N_{max}=60$ features, that gets a 24.5% of error.

**Figure 4:**
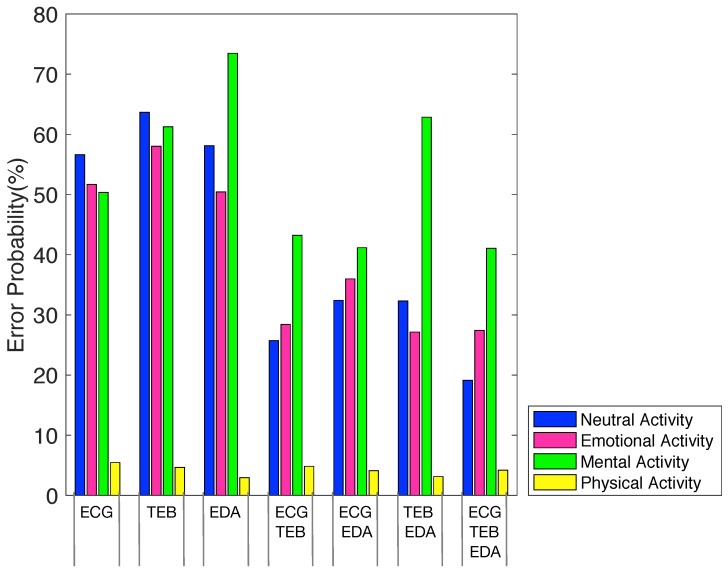
Error probability for each feature set and activity.

Figure 4 shows the error probability for each feature set and for each activity with the LSLC classifier and $N_{max}=40$ features, where it is possible to observe the percentages of error, being the lowest value obtained using all signals (ECG+TEB+EDA). Furthermore, we can appreciate that the activity most recognizable for all feature set is the physical activity and the least one the mental activity.

**Figure 5:**
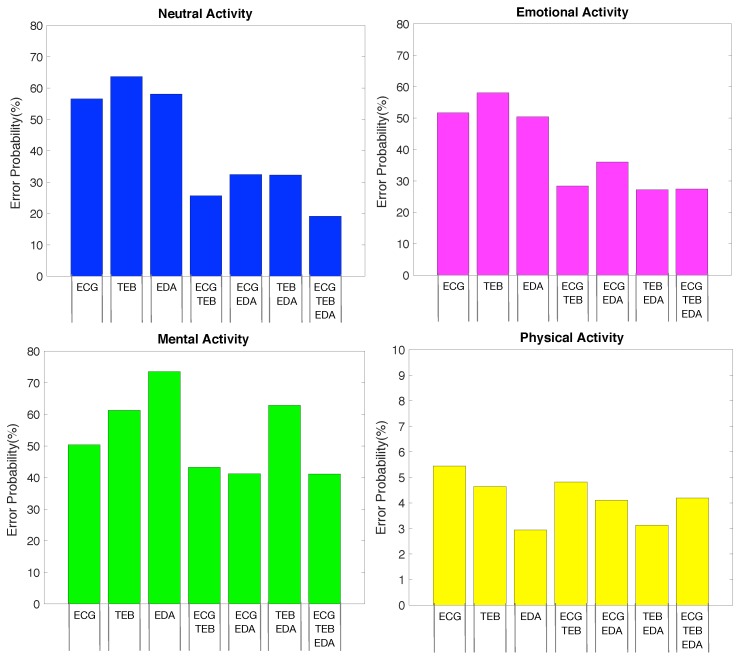
Error probability for each feature set and activity. Neutral (**top left**), Emotional (**top right**), Mental (**bottom left**) and Physical Activities (**bottom right**).

For a more detailed analysis, Figure 5 shows four different figures in which it is possible to observe each activity separately. The first one (top left) refers to the error probability for the neutral activity and for each of the feature set, where we can observe that the best performance of 19.11% is obtained using all feature set (ECG+TEB+EDA), provided by all signals. For the second one (top right) refers to the error probability for the emotional activity, in which the least error probability is 27.14% obtained for TEB+EDA. The third one (bottom left) shows the error probability for the mental activity, in which the minimum error probability is 41.07% using the feature set ECG+TEB+EDA. Finally, the fourth graph (on the bottom right) indicates the error probability for physical activity with errors ranging from 2.95% obtained with only EDA features to 5.45% for ECG. The error obtained for the ECG+TEB+EDA is 4.20%, which is very close to the minimum value.

On the other hand, if we analyze the signals separately, we can see that the independent signal which renders the best results is the TEB (29.50% with $N_{max}=40$ features and an MLP with 8 hidden neurons).

**Figure 6:**
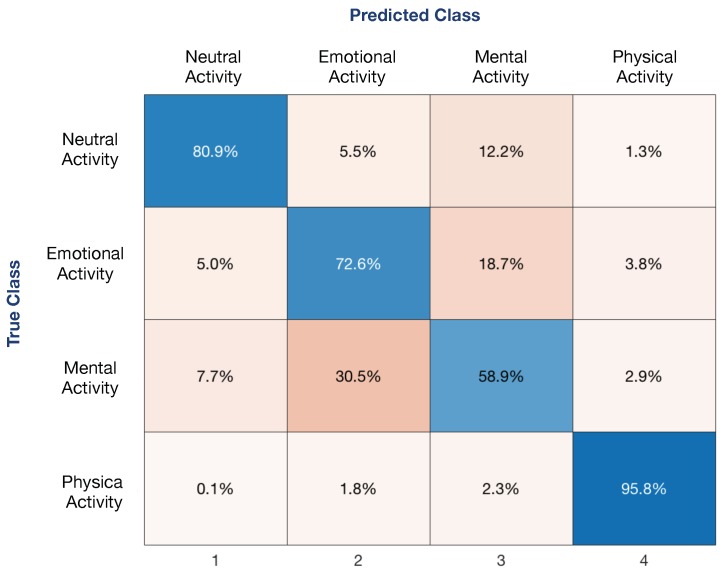
Confusion matrix between classes.

In order to study the main differences in the identification of the activity, the confusion matrix shown in Figure 6 indicates the misclassification between classes obtained using a LSLC and $N_{max}=40$ features obtained from all 3 signals (ECG+TEB+EDA), where the classes that present more misclassification are emotional and mental activity.

For a more detailed analysis of the performance of the classifiers when the number of features is varied, three figures are presented below. The figures represent the performance of the classifiers in the most significant cases. As with all features, it combines all feature sets. Another case, with the two signals that combined get the best result (ECG+TEB feature set), and the signal that gets the best result independently (TEB feature set).

**Figure 7:**
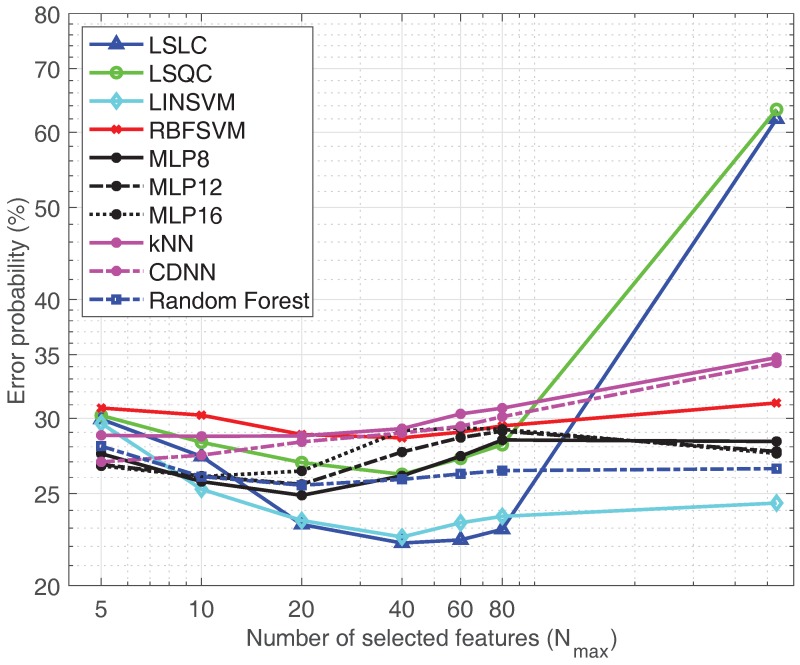
Classifiers comparison using *All feature set* (ECG+TEB+EDA).

Figure 7, presents the results obtained with the combination including all signals (ECG+TEB+EDA). We can see that the linear classifiers render the best results, and that the GA-based feature selection process that limits the number of features helps improving the performance of the classifiers. The fact that the complex classifiers (MLPs and RBFSVMs) do not match the results of the linear classifiers might imply the presence of strong generalization problems.

**Figure 8:**
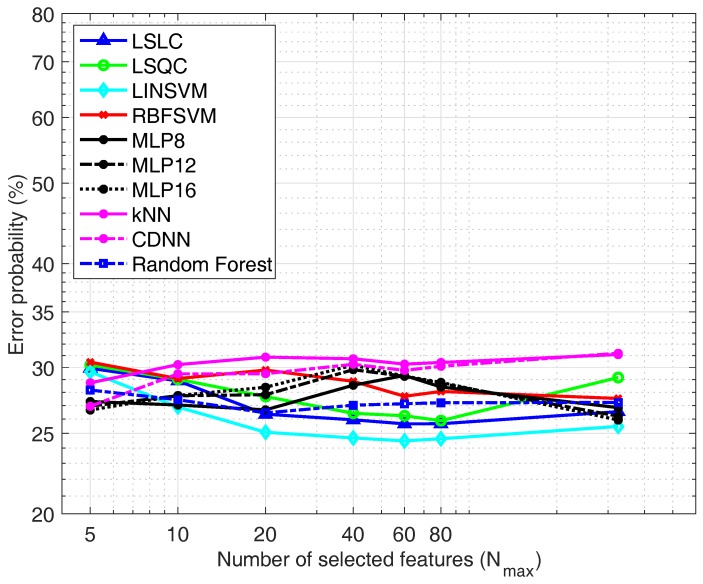
Classifiers comparison using the *ECG+TEB feature set.*

Figure 8 shows the performance of the classifiers when the ECG+TEB feature set is used. In this case again the best results are provide by linear classifiers. However, the classifier that renders the best result for ECG+TEB feature set is the LINSVM with an error probability of 24.5%.

**Figure 9:**
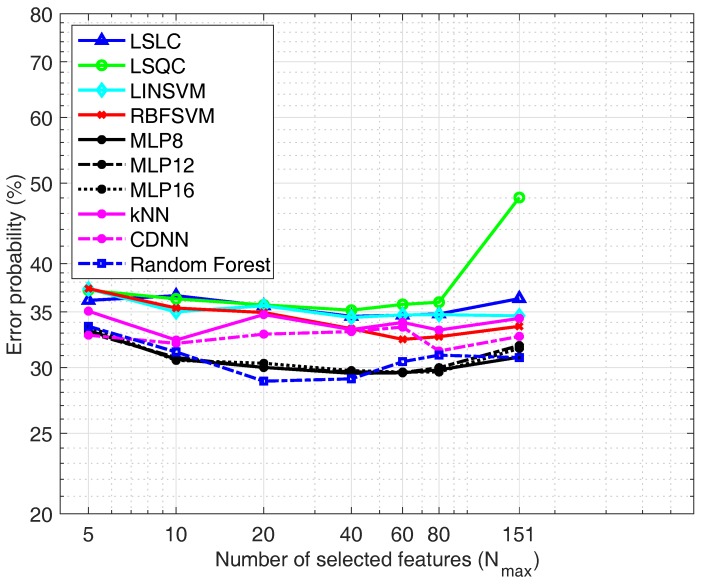
Classifiers comparison using the *TEB feature set.*

Finally, in case of considering just one signal the best choice is the use of the TEB. Figure 9 shows the performance of the classifiers under study with only features from the TEB signal. In this case the results are somewhat different from the previous ones, since the classifier that gives the best results is the RF, with an error probability of 28.9%.

### 5.3. Frequently Selected Features

In order to complete the study, we will show which features and measurements are the most frequently selected and the percentage of selection. Table 3 shows the average number of features selected by the GAs from each measurement and each signal, considering a maximum number of selected features $N_{max}=40$, for the different combination of signals. As we can see, the most frequently selected measurement from the ECG is the RR. In general, the measurements extracted in the frequency domain for the ECG are not very useful. Concerning the TEB, the RF and the BRV measurements present high ratios in the case of considering all the signals in the combination. And the most selected measurement from the EDA is the processed measurement taken in the hand.

To go deeper into the analysis, Table 4 shows the top-40 selected features, again in the case of selecting a maximum of $N_{max}=40$ features. In this case, we show the percentage of occurrence in the three best combinations of signals: the TEB alone, the TEB and the ECG, and the case of using all the biosignals. We can see that, in general, the mean baseline is one of the most frequent parameters. The most selected features from each signal in the case of considering all possible features in the GA are:

- From the ECG signal: the geometric mean of the HRV, the mean baseline of the RR, the logarithm of the SD of the RR, and the DFA1 of the HR.
- From the TEB signal: the average BR of the RF, the mean baseline of the BRV, and the minimum of the BRV.
- From the EDA measured in the hand: the mean baseline of the original measurement, and the mean baseline of the processed measurement.
- There are no features from the EDA measured in the hand which is used more than 40% of cases in the case of considering all possible biosignals in the GA. The most frequent one from this signal is the skewness of the processed measurement.

## 6. Discussion and Conclusion

Nowadays, activity recognition based on physiological signals is a relevant research field with a promising future. This paper presents an evaluation of the classification performance of different sensing modes ECG, TEB and EDA for detection of 4 different activities. The evaluation includes typical characterization features for the measured signal within each sensing mode. The characterization features included in the evaluation have been selected from a throughout review of the literature available. The evaluation has been done from several perspectives, the sensing mode perspective, the type of activity targeted and other parameters related to the feature extraction and classifier training. Consequently numerous conclusion can be derived from this work:

- In most of the relevant cases, the best results are obtained with a window length of 40 s. For the used database, the classifier that render the best results is the simplest ones, the LSLCs.
- When evaluating the combination of physiological signals which is better to correctly detect the type of activity, an LSLC trained with the feature set obtained when applying a GA considering all signals (TEB+ECG+EDA) achieves the lowest classification error probability (22.2%). In the case of the system trained with features selected from the ECG+TEB signal, the results are quite similar (24.5%), and there is no need to measure the EDA signal, making this choice very convenient for those cases in which we desire to pay attention to the simplicity of the acquisition system. That is, the comfort of the subject when there is no need to wear any glove or armband is higher, and the performance of the activity detection system is near the same.
- In addition, for each activity separately the feature set that provides the best results depends significantly on the activity under study. While for neutral activity and mental activity, the best result is obtained with ECG+TEB+EDA feature set, for emotional activity, the best result is obtained with TEB+EDA. Finally, the best result for physical activity is provided by the EDA feature set. This may be because the physical activity causes the activation of the sweat glands in a more meaningful way than the rest of the activities studied. In general, the signals working independently obtain worse results that when we make combinations between signals. Although it depends on the activity under study since in the case of physical activity the results are very similar using one or several signals. However, this does not happen in other cases in which the error is reduced in a remarkable way when combinations of signals are used in the training of the classifier. For the other type of activities, combining sensing modes provides similar or better performance than using only one type of sensing mode.
- The GA seems to be very useful in order to select the most relevant features, improving the results in terms of both complexity after training and error rate. From a total of 533 features, only 40 were necessary to achieve the minimum observed error. TEB signal seems to contain more useful information than the other signals.
- The results clearly suggest that the activity most easily identifiable is physical activity. Then the neutral, the emotional and finally the mental activity. This is due to the presence of misclassification between emotional and mental activities, as can be naturally expected.
- As a possible limitation of the study, we should consider that these conclusions might be different with other electronic devices. For instance, improvement on the textile based sensors or the use of gel-based classical sensors might improve the quality of the acquired signals, changing the usefulness of the measured features. Furthermore, the use of a more extensive database might overcome the generalization problems, allowing to obtain better results with more complex classifiers. In this sense, this paper does not try to propose a close solution but a methodology, and the comparison of the features and signals carried out might be conditioned to the actual textile sensor technology.

As a final conclusion, we have demonstrated the suitability of the GAs to select the best features among a wide dataset, containing most of the features identified as useful in the literature. The present study allows to extract significant conclusions concerning the information in each measurement, and determines a set of relevant measurements and features that can lead the research in future studies. On the other hand, the generalization capability of the classifiers has been identified as crucial in order to further improve the results in activity recognition through physiological signals, which opens new opportunities for researching within in the field.

## Figures and Tables

**Table 1:** Error Probability using a Least Squares Linear Classifier (LSLC) for the best number of features as function of the window length.

Combination of Signals	Par.	Window Length			
		10 s	20 s	40 s	60 s
ECG	Error(%)	43.0%	41.2%	40.1%	39.6%
	$N_{max}$	174	80	174	80
	*p*-value	<0.001	<0.001	<0.001	Best
TEB	Error(%)	51.0%	42.2%	34.6%	37.8%
	$N_{max}$	60	60	40	20
	*p*-value	<0.001	<0.001	Best	<0.001
ECG+TEB+EDA	Error(%)	26.6%	27.9%	22.2%	24.1%
	$N_{max}$	20	80	40	20
	*p*-value	<0.001	<0.001	Best	<0.001
ECG+TEB	Error(%)	41.9%	31.3%	25.7%	27.1%
	$N_{max}$	80	80	60	40
	*p*-value	<0.001	<0.001	Best	<0.001
ECG+EDA	Error(%)	26.0%	28.3%	27.9%	29.2%
	$N_{max}$	40	40	40	10
	*p*-value	Best	<0.001	<0.001	<0.001
TEB+EDA	Error(%)	29.9%	31.2%	29.7%	30.9%
	$N_{max}$	20	20	40	20
	*p*-value	<0.001	<0.001	Best	<0.001
EDA	Error(%)	36.1%	37.3%	36.5%	37.1%
	$N_{max}$	20	20	20	20
	*p*-value	Best	0.003	<0.001	<0.001

**Table 2:** Error probability (%) obtained for each classifier using the different combination of signals with a window length of 40 s.

Classifier		Single Signal				Combination of Signals				
						ECG				
		ECG	TEB	EDA	EDA	TEB	ECG	ECG	TEB	EDA
				Arm	Hand	EDA	TEB	EDA	EDA	
LSLC	Error	40.1	34.6	45.3	39.0	22.2	25.7	27.9	29.7	36.5
	$N_{max}$	174	40	10	5	40	60	40	40	20
LSQC	Error	39.3	35.2	71.2	52.8	26.2	25.9	40.6	31.9	51.4
	$N_{max}$	60	40	5	40	40	80	20	20	20
LINSVM	Error	41.0	34.5	61.7	47.0	22.5	24.5	36.4	28.7	47.2
	$N_{max}$	174	40	104	104	40	60	382	60	208
RBFSVM	Error	43.3	32.4	61.8	53.3	28.6	27.5	41.9	35.4	55.0
	$N_{max}$	174	60	40	40	40	325	80	20	40
MLP8	Error	41.3	29.5	61.7	43.9	24.9	26.7	35.8	29.2	46.4
	$N_{max}$	174	40	60	20	20	20	10	20	10
MLP12	Error	41.4	29.6	61.7	44.4	25.6	26.2	37.7	30.3	46.9
	$N_{max}$	174	60	60	20	20	325	10	20	10
MLP16	Error	41.6	29.6	61.9	45.1	26.1	25.9	38.2	30.5	47.3
	$N_{max}$	174	60	20	20	10	325	10	10	10
kNN	Error	45.6	32.4	55.4	49.0	28.7	28.7	40.3	33.1	50.5
	$N_{max}$	174	10	10	20	10	5	5	10	10
CDNN	Error	44.5	31.4	54.7	47.6	27.0	26.9	38.9	31.3	49.1
	$N_{max}$	174	80	5	10	5	5	10	20	10
RF	Error	41.0	28.9	54.9	50.9	25.5	26.5	36.7	28.2	46.5
	$N_{max}$	20	20	10	10	20	20	40	80	20

**Table 3:** Average number of features selected from the measurements of the different signals, with $N_{max}=40$ features.

	Single Signal				Combination of Signals				
					ECG				
	ECG	TEB	EDA	EDA	TEB	ECG	ECG	TEB	EDA
Signal: Measurement			Arm	Hand	EDA	TEB	EDA	EDA	
ECG: Original	6.5	-	-	-	1.5	3.5	3.8	-	-
ECG: RR	13.1	-	-	-	4.9	8.3	6.0	-	-
ECG: RA	6.7	-	-	-	2.4	3.5	2.2	-	-
ECG: HR	6.5	-	-	-	1.5	2.9	1.1	-	-
ECG: HRV	2.8	-	-	-	2.4	2.4	2.2	-	-
ECG: PSD	0.6	-	-	-	0.4	0.7	0.3	-	-
ECG: PSD-VLF	0.5	-	-	-	0.3	0.4	0.7	-	-
ECG: PSD-LF	0.6	-	-	-	0.4	0.5	0.8	-	-
ECG: PSD-MF	0.9	-	-	-	0.4	0.6	0.9	-	-
ECG: PSD-HF	1.0	-	-	-	0.4	0.7	0.7	-	-
ECG: PSD-VLLF	0.6	-	-	-	0.3	0.4	0.7	-	-
TEB: Original	-	8.4	-	-	1.4	3.1	-	1.6	-
TEB: LF	-	8.9	-	-	1.4	3.8	-	1.5	-
TEB: RF	-	10.1	-	-	2.0	1.2	-	3.3	-
TEB: BRV	-	5.0	-	-	2.5	3.6	-	3.8	-
TEB: PSD	-	1.9	-	-	0.3	0.6	-	0.2	-
TEB: PSD-VLF	-	0.9	-	-	0.6	0.5	-	0.7	-
TEB: PSD-LF	-	0.8	-	-	1.0	1.0	-	1.1	-
TEB: PSD-MF	-	1.0	-	-	1.0	0.9	-	1.1	-
TEB: PSD-HF	-	2.3	-	-	0.7	0.9	-	0.6	-
TEB: PSD-VLLF	-	0.8	-	-	0.6	0.6	-	0.7	-
EDA-arm: Original	-	-	5.6	-	0.6	-	1.6	1.3	2.6
EDA-arm: Processed	-	-	9.8	-	1.3	-	2.4	2.5	3.5
EDA-arm: LF	-	-	8.9	-	0.6	-	1.5	1.3	4.0
EDA-arm: HF	-	-	7.8	-	0.6	-	0.8	0.8	3.8
EDA-arm: PSD	-	-	3.2	-	0.5	-	0.9	0.7	1.4
EDA-arm: PSD-LF	-	-	1.9	-	0.4	-	0.4	0.7	0.5
EDA-arm: PSD-HF	-	-	2.9	-	0.4	-	0.8	0.6	1.3
EDA-hand: Original	-	-	-	2.8	1.9	-	1.7	2.1	2.3
EDA-hand: Processed	-	-	-	11.6	3.9	-	6.0	6.9	9.2
EDA-hand: LF	-	-	-	6.1	1.5	-	1.5	1.9	2.7
EDA-hand: HF	-	-	-	6.6	1.4	-	1.7	2.0	3.2
EDA-hand: PSD	-	-	-	1.3	0.2	-	0.4	0.7	0.8
EDA-hand: PSD-LF	-	-	-	7.1	0.2	-	0.6	2.4	3.1
EDA-hand: PSD-HF	-	-	-	4.6	0.2	-	0.4	1.2	1.6

**Table 4:** Top-40 selected features from the different signal, and percentage of occurrence with $N_{max}=40$ features.

Feature			Combination of Signals		
					ECG
			TEB	ECG	TEB
Signal	Measure	Parameter		TEB	EDA
TEB	RF	Average BR	100%	0%	100%
TEB	BRV	Mean baseline	100%	0%	100%
EDA-hand	Original	Mean baseline	0%	100%	100%
EDA-hand	Processed	Mean baseline	0%	100%	100%
ECG	HRV	Geom. mean	0%	0%	100%
ECG	RR	Mean baseline	0%	0%	100%
ECG	RR	log(SD())	0%	0%	99%
ECG	RR	DFA1	0%	0%	98%
TEB	BRV	Minimum	100%	0%	94%
ECG	HR	Mean baseline	0%	0%	93%
ECG	HRV	Mean baseline	0%	0%	87%
ECG	RA	Mean baseline	0%	0%	68%
EDA-hand	LF	Mean baseline	0%	43%	56%
TEB	PSD-VLLF	Mean baseline	66%	0%	50%
TEB	PSD-MF	Mean baseline	97%	0%	50%
EDA-hand	Processed	Number SCR	0%	100%	49%
EDA-hand	HF	Mean baseline	0%	57%	48%
TEB	PSD-VLF	Mean baseline	72%	0%	48%
TEB	PSD-LF	Mean baseline	56%	0%	48%
ECG	Original	Skewness	0%	0%	44%
ECG	RA	Mean abs. dev.	0%	0%	40%
EDA-arm	Processed	Skewness	0%	8%	40%
TEB	PSD-HF	HF/LF	78%	0%	39%
TEB	LF	Mean baseline	100%	0%	37%
ECG	RA	SD	0%	0%	36%
TEB	Original	Mean baseline	100%	0%	36%
ECG	RR	25% Trm. mean	0%	0%	36%
TEB	PSD-LF	(LF+MF)/HF	25%	0%	35%
EDA-hand	Processed	PNS	0%	35%	35%
EDA-hand	Processed	NZC	0%	41%	34%
EDA-hand	Processed	PZC	0%	24%	33%
TEB	PSD-MF	MF/HF	5%	0%	33%
TEB	RF	Mean baseline	100%	0%	33%
TEB	LF	Percentile 75%	93%	0%	32%
EDA-hand	Processed	Maximum	0%	82%	31%
ECG	RR	Median	0%	0%	31%
EDA-hand	Processed	Minimum	0%	47%	27%
EDA-hand	Processed	Median	0%	100%	26%
ECG	RR	Geom. mean	0%	0%	25%
TEB	Original	Percentile 75%	16%	0%	23%

**Figure A1:**
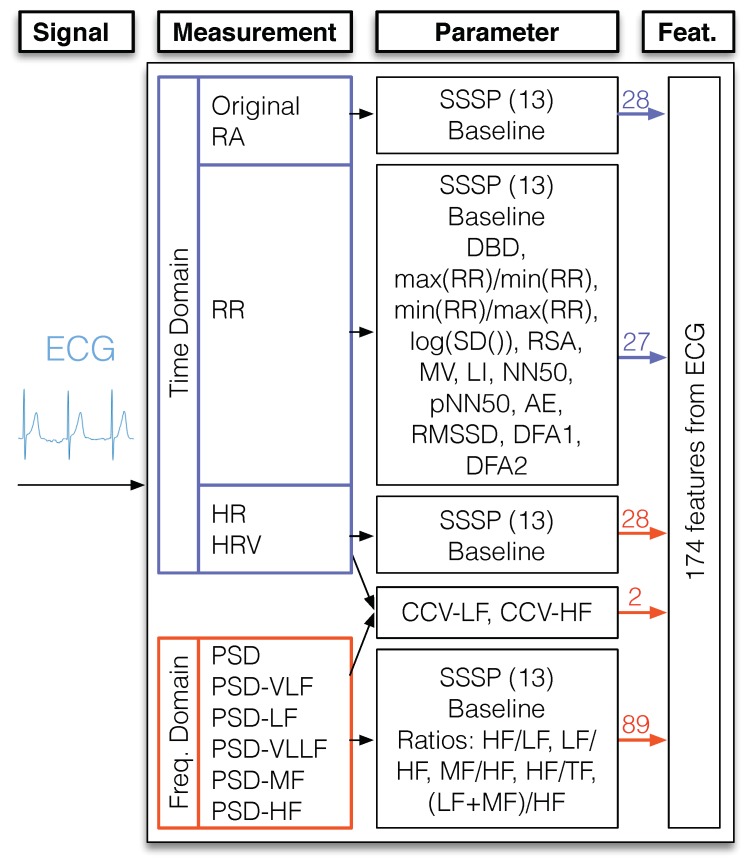
ECG-based feature extraction scheme.

**Figure A2:**
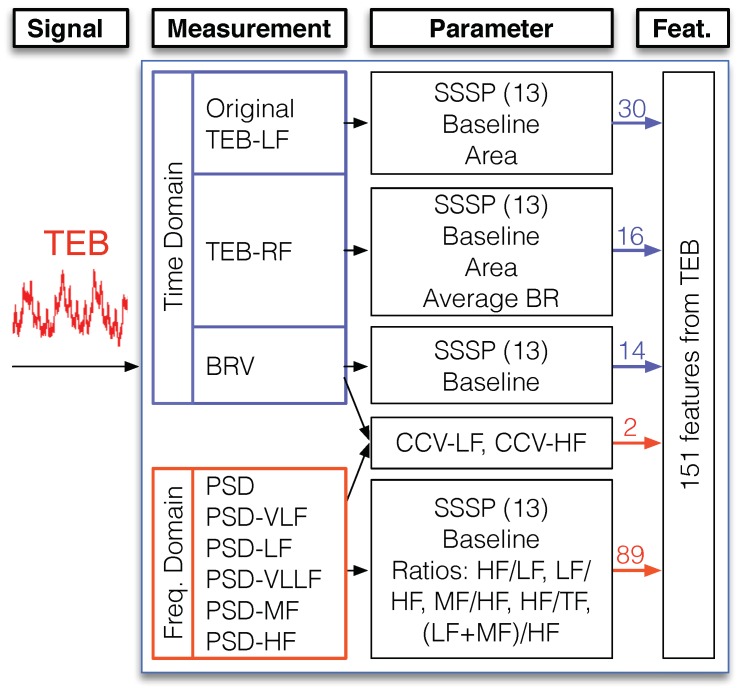
TEB-based feature extraction scheme for the classical set of features.

**Figure A3:**
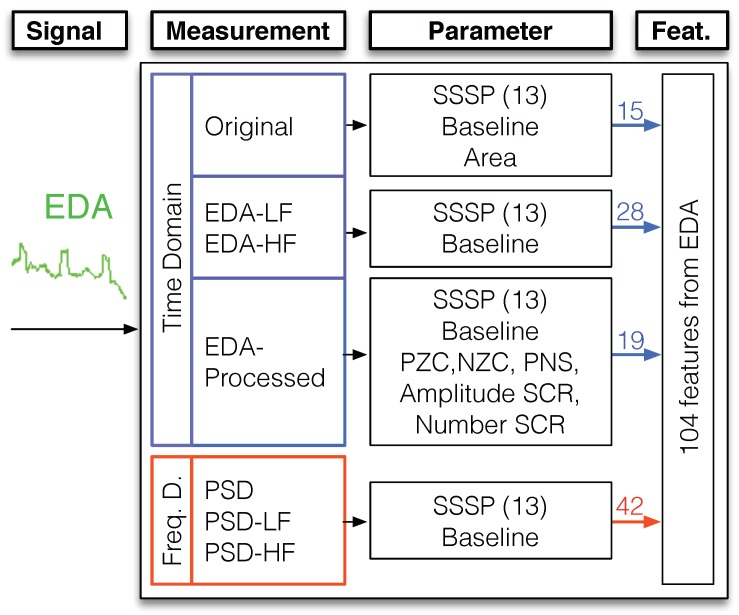
EDA-based feature extraction scheme.

## Supplementary Materials

The following are available online at https://www.mdpi.com/1424-8220/19/24/5524/s1, Supplementary Data.

## Abbreviations

The following abbreviations are used in this manuscript:

ECG	Electrocardiogram
TEB	Thoracic Electrical Bioimpedance
EDA	Electrodermal Activity
HRV	Heart Rate Variability
SDNN	Standard Deviation of NN intervals
SDSD	Standard Deviation of Successive Differences
LSLC	Least Squares Linear Classifier
LSQC	Least Squares Quadratic Classifier
SVM	Support Vector Machine
LINSVM	Linear Support Vector Machine
RBFSVM	Radial Basis Function Support Vector Machine
MLP	Multi-Layer Perceptron
kNN	*k*-Nearest Neighbor
CDNN	Centroid Displacement-based Nearest Classifier
RF	Random Forest
GA	Genetic Algorithm
SSSP	Standard Set of Statistical Parameters

## Appendix A. Features from the ECG Signal

The measurements used to characterize the ECG can be divided into two groups, those calculated in the *time domain* and those calculated in the *frequency domain*. Figure A1 shows the features extracted from the ECG signal. As we can appreciate, there are 83, 89 and 2 features for the time domain, frequency domain, and the mixed domain, respectively. That means that the total number of features calculated to characterize the ECG measurement is 174.

### Appendix A.1. Time-Domain

A total of five measurements were considered in the time-domain, directly or indirectly extracted from the QRS analysis:

- The original unprocessed signal [53,55] and the R wave Amplitude (RA) (amplitude of the different R waves in each window). The SSSPs and the baseline parameters were calculated for these measurements.”
- The interval between successive Rs (RR) (time lapsed between successive R waves) [35,49,80]. Apart from the SSSP and the baseline, some special features have been extracted from the RR measurement:
  – Deep Breathing Difference (DBD), calculated as the difference between the maximum RR and minimum RR in the window under study [81,82].
  – Ratios maximum RR vs. minimum RR, that is, RRmax/RRmin and RRmin/RRmax [83].
  – Logarithm of the standard deviation of RR in the window under study.
  – Respiratory Sinus Arrhythmia (RSA), calculated as the quotient between the DBD and the mean value of the RR in the window under study. This measurement is related to the function of parasympathetic nervous during spontaneous ventilation [84].
  – Modal Value (MV), defined as the most frequent value in the RR intervals in the window under study [40].
  – Load Index (LI), based on the ratio between the number of occurrences of each Modal Value and DBD [40].
  – NN50, determined as the number of successive RR interval pairs differing by more than 50 ms [40,42,48].
  – pNN50, obtained dividing NN50 by the total number of RR intervals [40,42,48].
  – Approximate Entropy (AE), originally proposed in [85], and applied to physiological data in [39,42,86].
  – Root Mean Square of Successive Differences (RMSSD), determined by calculating the square root of the mean squared difference between consecutive RR intervals [34,48,49,87]. The RMSSD is the primary time domain used to estimate the high-frequency beat-to-beat variations that represent vagal regulatory activity [48].
  – Two parameters of the Detrended Fluctuation Analysis (DFA) [85]. These two parameters (DFA1 and DFA2) have been used to quantify the presence or absence of fractal-like correlation properties of the heart period time series [39].
- Heart Rate (HR). It is measured as the number of pulses per unit of time, usually beats per minute (bpm). It is calculated as the inverse of the RR interval. It is obtained through the inverse of the RR interval. This parameter is highly important, as it is related to physical exercise, anxiety, sleep, illness, food intake, and drugs, among others. The increase or decrease on this speed is the answer of our body or mind condition [34,48]. The SSSPs and the baseline parameters were calculated from this measurement.
- Heart Rate Variability (HRV), which has been widely used to extract information about the status of the autonomic nervous system and emotions [23]. The work [88] provides a review of this measurement. In addition, numerous studies reveal the importance of this parameter [23,26,38,39,41,42,45,48,50,51,89]. We decided to obtain the HRV as proposed in [26], where the HRV is determined from a modified version of the HRV sampled at 256 Hz. Once the HRV is obtained, it is possible to extract different valuables features, using the SSSPs and the baseline parameter.

Many other measurements were found in the literature such as SDNN index, SDANN among others [34]. However, we did not use these measurements because they require at least 5 min to be calculated, since they are often calculated over a 24-h period.

### Appendix A.2. Frequency-Domain

The other main group of measurements are evaluated in the frequency domain, through the Discrete Fourier Transform (DFT). The main measurements taken in this part were:

- Power Spectral Density (PSD) of the HRV signal is obtained using Welch’s method [23,38,42].
- Power per Bands. From this PSD parameter, several frequency bands were considered: Very-Low-frequency (PSD-VLF), taken from 0.0033–0.05 Hz; Low Frequency (PSD-LF) from 0.05–0.08 Hz; Very-Low and Low-Frequency (PSD-VLLF) from 0.0033–0.08 Hz, Mid Frequency (PSD-MF) from 0.08–0.15 Hz. and High frequency (PSD-HF) from 0.15–0.5 Hz. These values were established taking into account several papers such as [23,26,34,36,37,38,40,41,42,43,44,45,46,47,48].

The parameters taken from these spectral measurements were the SSSPs, the baseline parameter, and a set of specific parameters related to ratios between the average power for the different bands: HF/LF, LF/HF, MF/HF, (LF+MF)/HF, and HF/TF, being TF the total power in all frequencies [23,43,44,46,47,49].

### Appendix A.3. Mixed Domain

There were also two parameters taken from relationships between time and frequency parameters, denoted as Coefficients of Component Variance (CCV). The CCVs considered were the CCV-LF and the CCV-HF [39], and they were calculated as the square root of LF or HF power divided by the average HR.

## Appendix B. Features from the TEB Signal

The measurements used in order to extract the most relevant information from TEB signal follow a structure similar to the one described in the case of the ECG signal, being again divided into the time domain, frequency domain, and mixed domain features.

Figure A2 shows the features extracted from the TEB signal. There are 60 time domain features, 89 frequency domain features, and 2 mixed domain features. Therefore, the total number of features calculated to characterize the TEB signal is 151.

### Appendix B.1. Time Domain

- TEB-Original Signal: The 13 SSSPs and the baseline parameter aforementioned are calculated from the TEB-Original signal. Apart from these parameters, the area was also calculated, using an approximated segment-based integral of the measurements via a trapezoidal method with unit spacing.
- TEB-LF: the original signal is low-pass filtered (LF block) with a cutoff frequency of 3 Hz, using an FIR filter with order $N_{1}=100$. Again, the 13 SSSPs and the baseline parameter are calculated.
- TEB-RF: Additionally, another new signal is obtained from TEB-LF. The first low pass filter (LF block) acts as an anti-aliasing filter, which allows the use of Interpolated Finite Impulse Response (IFIR) filters [90]. Thus, the output of this anti-aliasing filter is applied to a band-pass filter with cutoff frequencies of 0.1 Hz and 0.5 Hz with a stretch factor of SF=10 and an order $N_{2}=5\times F_{TEB}-N_{1}=400$, (being $F_{TEB}=100$ Hz). We denominate TEB Respiration Frequency (TEB-RF) to the measurement obtained. The TEB-RF measurement was used to determine the Breathing Rate (BR). This parameter calculates the number of breaths per minute [91] using a peak detection algorithm. The parameters taken from this measurement, apart from the SSSPs, the baseline, and the area, include the average BR.
- Breath Rate Variability (BRV). Using the BR measured from the TEB-RF, we can calculate the Breath Rate Variability (BRV) in a similar way to HRV. The 13 SSSPs and the baseline parameter are calculated from this measurement.

### Appendix B.2. Frequency Domain

The frequency features calculated from TEB are similar to those calculated from ECG. So, again the PSD of the original signal was calculated and applied to several filtered versions of the signal. Again, apart from standard parameters, power ratios were also evaluated.

### Appendix B.3. Mixed Domain

The features considered are the CCV mixed-domain parameters (in this case, using the average BR to normalize the squared root of the energy).

## Appendix C. Features from the EDA Signal

The structure used in order to extract the most relevant information from the EDA signal follows a structure similar to the one described in the case of the ECG signal and the TEB signal.

Figure A3 shows the measurements obtained from the EDA signal and the procedure used to extract the features. In total, we obtained 62 time domain features and 42 frequency domain features. Taking into account that the EDA was registered in both the hand and the arm (as was described above), we obtained 104 features from the arm EDA and 104 features from hand EDA.

### Appendix C.1. Time Domain

We obtained four different time domain measurements:

- EDA-Original Signal. The 13 SSSPs and the baseline aforementioned parameter are calculated to the EDA-Original signal. The area is also calculated from this measurement, using an approximated integral of the time segments through a trapezoidal method with unit spacing.
- EDA-LF: The original signal is filtered with a 20-order low-pass FIR filter (LF block) with a cutoff frequency of 0.2 Hz [41]. The 13 SSSPs and the baseline parameter were evaluated.
- EDA-HF: A complementary filter is also applied to obtain the high frequency components (20-order high-pass FIR filter with a cutoff frequency of 0.2Hz), and the same parameters than those from the EDA-LF measurement are evaluated over the obtained EDA-HF measurement.
- EDA-Processed: The work [26] shows the steps to process EDA signal, for Skin Conductance Response (SCR) detection. The process consists in removing the mean value, resampling to 20 Hz, time differentiating, and filtering with a 20-order Bartlett window. From the processed EDA measurement, typical parameters are extracted using the SSSPs and the baseline parameter, and also some specific parameters:
  – Zero Crossing (ZC): Positive ZC (PZC), and Negative ZC (NZC) are interesting for the application at hand [26,41].
  – Ratio or proportion of Negative Samples (PNS), evaluated as the quotient between the number of negative samples and the total number of samples [41].
  – SCRs were evaluated analyzing the zero crossings in the processed EDA signal. The average amplitude of the SCR occurrences and the number of occurrences in the analysis window were used as parameters [26,42,54,91]. SCRs were determined by finding two consecutive zero-crossings, from negative to positive and from positive to negative.

### Appendix C.2. Frequency Domain

The PSDs extracted from the EDA-Original signal, the EDA-LF and the EDA-HF, were taken as spectral measurements, using a Welch’s overlapped segment averaging estimator. The SSSPs and the baseline parameter were calculated from these measurements.
